# Opinion: The Key Steps in the Origin of Life to the Formation of the Eukaryotic Cell

**DOI:** 10.3390/life14020226

**Published:** 2024-02-05

**Authors:** Clifford F. Brunk, Charles R. Marshall

**Affiliations:** 1 Department of Ecology and Evolutionary Biology, University of California Los Angeles, Los Angeles, CA 90095-1606, USA; 2 Department of Integrative Biology and Museum of Paleontology, University of California, Berkeley, CA 94720-4780, USA

**Keywords:** origin of life, alkaline hydrothermal vent microchambers, prebiotic chemistry, acetyl-CoA pathway, RNA world, LUCA, chemiosmosis, ATP synthase, archaea, bacteria

## Abstract

The path from life’s origin to the emergence of the eukaryotic cell was long and complex, and as such it is rarely treated in one publication. Here, we offer a sketch of this path, recognizing that there are points of disagreement and that many transitions are still shrouded in mystery. We assume life developed within microchambers of an alkaline hydrothermal vent system. Initial simple reactions were built into more sophisticated reflexively autocatalytic food-generated networks (RAFs), laying the foundation for life’s anastomosing metabolism, and eventually for the origin of RNA, which functioned as a genetic repository and as a catalyst (ribozymes). Eventually, protein synthesis developed, leading to life’s biology becoming dominated by enzymes and not ribozymes. Subsequent enzymatic innovation included ATP synthase, which generates ATP, fueled by the proton gradient between the alkaline vent flux and the acidic sea. This gradient was later internalized via the evolution of the electron transport chain, a preadaptation for the subsequent emergence of the vent creatures from their microchamber cradles. Differences between bacteria and archaea suggests cellularization evolved at least twice. Later, the bacterial development of oxidative phosphorylation and the archaeal development of proteins to stabilize its DNA laid the foundation for the merger that led to the formation of eukaryotic cells.

## 1. Introduction

The literature on the origin of life is vast and growing. As is usually the case for complex problems, most publications focus on specific aspect of the problem. Here, we take a step back and present our view of the overall path, from the initial steps that led to the origin of life all the way to the emergence of the eukaryotic cell, as well as providing some historical context. We undertake this effort in full recognition of the fact that we do not yet have a comprehensive understanding of all steps in this path, and that many will disagree with aspects of our narrative. Nonetheless, we feel that there is value in attempting to sketch out the whole path, in part because it is rarely undertaken, in part because it may be of value to researchers and teachers interested in the full sweep of major innovations that led to the emergence of eukaryotes, and in part because sometimes coherency can emerge from a broader view.

We do not provide an encyclopedic review of the vast literature on this set of transitions, but we hope that our citations (and the references therein) will provide a useful introduction to this literature. Finally, no disrespect is intended toward other perspectives and scenarios than those chosen here—our objective is simply to produce an outline of the most plausible scenario given our reading of the information available.

### 1.1. Some Guiding Principles

Before getting into our narrative, we outline some of the principles and assumptions that have guided our thinking, especially with regard to the early phases of life’s origin.

#### 1.1.1. Steady and Substantial Fluxes

Life today depends on substantial and reliable fluxes of energy and raw materials. The fact that organisms are open systems and that the timescales of sustaining biochemical reactions are relatively fast (seconds not months) means that organisms cannot survive long in the absence of these sustaining resources. Given this dependency, and that first life was likely relatively inefficient compared to its living descendants from which our knowledge of biochemistry derives, we feel that the initiation of life must have occurred in an environment with substantial and reliable fluxes of energy and raw materials. Thus, it is not sufficient to show that certain conditions can lead to the generation of key molecules; it is also necessary that those conditions were maintained by long-term and substantial flows of energy and raw materials.

#### 1.1.2. QWERTY and the First Metabolism

In the context of this paper, the QWERTY ‘principle’ refers to the fact that the current state of an evolving system typically retains evidence of its history. The principle is also used by evolutionary biologists to capture the idea that as a consequence of this historical path dependence, some current functions will be suboptimal. The name stems from the fact that the layout of the keyboard today, with the first row of letters beginning with QWERTY, is largely a consequence of the initial design [1,2], which, while perhaps optimal at the time [3], appears to be now less efficient than other designs [4].

Living organisms are replete with QWERTY-like legacies of their past; for example, the fact that some aerobic metabolic pathways (e.g., for sterol and fatty-acid synthesis) are built on anaerobic foundations [5], the fact that the catalytic function of the ribosome, peptidyl transferase, is a ribozyme, as well as the fact that the enzyme that assimilates CO_2_ during photosynthesis, RuBisCO, is both slow and, as it turns out, has an oxygenation function (photorespiration) that competes with the carbon-fixing function of the Calvin cycle at high temperatures and in dry environments [6]. We say ‘turns out’ because there was no free oxygen when RuBisCO was first used in bacterial carbon fixation [7] and thus photorespiration was not a selective issue at the time.

In the context of the origin of life, of the six known CO_2_ fixation pathways among living bacteria and archaea, only one, the acetyl-CoA pathway, is shared by both groups [8]. It is also the only linear pathway (and thus the only one without the stereochemical constraints of cyclic pathways) that generates rather than requires energy (it is exergonic) [8]. It uses enzymes that have at their cores transition metal clusters found in the hydrothermal vents, and is strictly anaerobic (consistent with it being ancient) [8,9]. Taken together, this evidence strongly suggests that the acetyl-CoA pathway was the first CO_2_ fixation pathway [8]. Thus, all else being equal, we favor origin-of-life scenarios that can account for its early origin; the acetyl-CoA pathway is a prokaryotic QWERTY. In contrast, scenarios that imply that the first organisms had a metabolism completely unknown today, while possible, seem less plausible because it is hard to see how such a metabolism (for example, a pathway driven by UV and wet-dry cycling) could have been lost without leaving a trace of its existence in the biochemistry of any living species.

#### 1.1.3. Pre-Adaptation

Epistemologically, the study of the origin of life is unique because we cannot use living organisms to bracket the emergence from non-life to life. In contrast, there are living taxa that bracket all other major evolutionary transitions, i.e., there are extant species that both predate and postdate each transition. For example, for the origin of the eukaryotic cell, we have the bacteria and archaea that predate the transition and many single celled eukaryotes that postdate the transition.

An analysis of life’s major transitions indicates that they were: (1) often built upon extensive pre-adaptations, where much of the innovation had already evolved for some other function typically millions of years or more prior to the time of the transition itself; (2) that the transitions were often dependent on, or selected for, by environmental changes driven by other lineages. For example, the vertebrate invasion of land was built upon the pre-adaptive origin of limbs, digits and lungs that had evolved for life in water [10,11], and the transition was not possible until there was a terrestrial ecosystem in place, including plants [12], fungi and bacteria [13], and invertebrates [14]. Similarly, the Cambrian explosion, the rapid appearance of the majority of the animal phyla [15], was built upon extensive genomic preadaptation [16], including the evolution of a combinatorial developmental system [15,17]. Further, the Cambrian explosion occurred only after atmospheric oxygen levels had risen substantially [15,18]. The origin of the modern eukaryotic cell was dependent on the origin of histone-like proteins in the archaeal partner [19], and oxidative phosphorylation in the bacterial partner [20,21], which itself was a response to the rise in oxygen, which in turn was as a consequence of the emergence of oxygenic photosynthesis, which depended on the origin of anoxygenic photosynthesis, etc.

Thus, it seems likely that the emergence of life was also built on pre-adaptations (or the non-evolutionary equivalent of preadaptation). Candidates include DNA, whose role as the primary information storage molecule may have built upon earlier roles, for example, to help stabilize RNAs [22]. Another is the probability that protein-like molecules, proteinoids, may have been an integral part of the pre-biotic chemistry, for example by providing hydrophobic pockets needed in alkaline hydrothermal vents for the formation of the hereditary materials, RNA and DNA. Similarly, it seems likely that lipids also formed early, playing a critical role in determining which molecules entered the vent microchambers where the pre-biotic chemistry was being driven by the external energy and material fluxes, prior to their role as the primary containment for life’s internal processes. In this context, there is evidence that interactions between the inorganic walls of the vent microchambers and fatty alcohols may have facilitated the process of providing containment for life’s early chemistry [23]. Even RuBisCO may have evolved for another function prior to its role in photosynthesis in bacteria [7]. Finally, well after life originated, the electron transport chain used to pump hydrogen for the use of ATP synthase was co-opted as part of the photosynthetic apparatus [24].

Having said this, many evolutionary innovations have also arisen by direct adaptation, where complexity arose from the persistent action of direct selection. For example, this was probably true for complex eyes, which have evolved multiple times, in vertebrates, squid and their kin, as well as arthropods. Similarly, an emerging theory for the origin of the ribosome, the machinery that makes proteins, suggests that its complexity arose directly from a co-evolutionary process, rather than via the co-option of previously evolved complexity [25,26,27].

#### 1.1.4. The Likely Complexity of Life’s Evolutionary Path

An implicit assumption in some of the origin of life literature is that the emergence of life was a relatively straightforward process [28,29,30]. The experiments of Sutherland, where RNA, protein, and lipid precursors are generated simultaneously seem to support this notion [31,32], although Sutherland argues that the path to first life was complex [33]. The process could have been relatively straightforward, but it seems more likely that the process was very complex. The principle of preadaptation suggests that the path was a drawn-out multi-step process, with key steps only becoming possible after the development of earlier innovations.

An important implication of this proposition is that it is unlikely that we will ever be able to replicate the origin of life via simple chemical experiments; we suspect the origin of life was not a ‘one-pot’ process. The notion of preadaptation suggests that as pre-biotic chemistry developed, new chemical regimes emerged which made once impossible reactions now possible. We argue that spatial heterogeneity was key to diversifying the range of chemical regimes upon which life relies on today, and thus needed to develop as life emerged [34].

The view that there were secular changes in the chemical regimes as the pre-biotic chemistry developed is particularly important. For example, some argue that life could not have originated in undersea hydrothermal vents because both alkalinity and aqueous environments dissociate nucleic acids, the so-called ‘water problem’ [35]. It has been stated based on these considerations “*that the idea that life originated at vents should, like the vents themselves, remain ‘In the deep bosom of the ocean buried’*” [33]. However, this has been forcefully rebutted by Russell [36].

Today, a great deal of chemistry occurs in living cells that is incompatible with both the cell’s external chemical regime and with its internal chemical regime. For example, the polymerization of amino acids into proteins and nucleotides into DNA and RNA occur in cells that are 70%, by weight, water. It seems entirely reasonable, for example, that as the pre-biotic chemistry developed, the chemical regimes inside the proto-organism became increasingly heterogenous, and that hydrophobic pockets developed where the polymerization of amino acids into proteinoids and nucleotides into nucleic acids could occur. If core elements of life originated very rapidly, then there may be a ‘water problem’ with the alkaline hydrothermal vent hypothesis. However, if the path were more complex, then in principle the problem for this scenario disappears.

#### 1.1.5. The Application of Guiding Principles

We assume that life most likely arose in alkaline hydrothermal vents. However, others favor the idea that life started on the edge of terrestrial hot springs, where the initial components (amino acids, lipids, precursors of nucleotides) were formed under oscillating wet–dry conditions using UV light as the energy flux and HCN as the key food stock [27,28,29,30,31,32,37]. However, this hypothesis does not satisfy the four principles outlined above as well as the alkaline hydrothermal vent hypothesis and, in some cases, does not at all. The alkaline hydrothermal vent setting has voluminous long-lived fluxes of energy and raw materials; it quite remarkably predicts that the first metabolic pathway was an abiotic equivalent of the Acetyl-CoA pathway. It incorporates the idea of spatial heterogeneity and thus embraces the principle of preadaptation. This setting allows the path to the origin of life to be protracted, in part because it does not depend on proto-life having to be spatially autonomous from the outset. The hypothesis is also attractive given the vast number of microchambers in a given locality [34] and the possibility that the driving geochemistry, serpentinization [8,38], may have been especially widespread in the Hadean, when life evolved [34]. Note that the alkaline hydrothermal vent scenario actually requires the notion of protracted development, given that alkaline aqueous environments are inimical to the formation of RNA and DNA.

In contrast, the wet–dry cycling hypothesis is not in an environment with consistent large-scale continuous fluxes of raw materials, does not predict the nature of the first metabolism, leads to the conclusion that none of the early steps in the origin of life have left their signature in the living biota, and fails to come up with a plausible hypothesis for how a long-term stepwise, path-dependent, emergence of life occurred. Further, unlike the alkaline hydrothermal vent hypothesis, it fails to explain any of life’s current idiosyncrasies: for example, the differences in the compositions of the cell walls, cell membranes, and locomotory devices of bacteria and archaea, and the bizarre nature of life’s use of chemiosmosis in the generation of ATP [34]. So, with this lengthy preamble, we now turn to our narrative.

### 1.2. A Short History of Our Current Understanding

By the dawn of the 21st century, all the essential information needed for assembling a plausible scenario for the origin of life on Earth was in place [39]. This process began in earnest in English early in the 20th century, with the speculations of Alexander Oparin (1894–1980), John Burdon Sanderson Haldane (1892–1964), and others [40,41,42], but there is an older literature in German [8,43,44]. The first experimental data were generated by Miller and Urey in 1953, showing that multi-carbon molecules including amino acids are easily generated [45]. The discovery of ribozymes, catalytic RNAs, in 1980 by Thomas Cech (1947–) showed that RNA is capable of catalyzing reactions as well as transmitting information [46]. This broke the impasse over the origin of DNA and proteins, given their co-dependent generation. Then, in 2000, the discovery of the ‘Lost City’ alkaline hydrothermal vents by Deborah Kelley (1958–) and colleagues provided an attractive present-day analog of the venue for the origin of life [47,48]. Since that time, fueled in part by the broad interest in astrobiology, the origin-of-life literature has grown enormously.

In light of these advances, we survey both the history and current understandings of life’s path from its inception to the origin of the eukaryotic cell. Our intent is to provide a sweeping overview rather than a detailed description of each specific phase. We begin with what appears to us to be the most plausible scenario for the origin of life, but we note that this plausible scenario is just that—it is not the ‘truth’, and may be shown to be inadequate in one or more ways. Camprubí et al. provide a good summary of the range of proposed scenarios [49].

The events relevant to the origin of life transpired in the distant past, probably more than 4 billion years ago. The oldest water-lain sediments date to 3.8 to 3.9 billion years ago [50], so there is virtually no geologic data from the time of life’s origin [51,52]. While the exact environment on Earth at that time is less than certain [49], it is clear that the young Earth was very different, including being effectively devoid of atmospheric molecular oxygen [18] and life. However, the underlying principles of physics and chemistry clearly applied to the Earth at the time life began, as they do today, and thus provide a framework for understanding the earliest phases of life’s history.

### 1.3. The Definition of Life

A well-used definition of life comes from NASA: ‘Life is a self-sustaining chemical system capable of Darwinian evolution’ [53]. In some respects, this definition is incomplete, as it depends on an understanding of Darwinian evolution. As an aside, Darwin’s actual understanding of evolution is different from ours today, in part because he believed that heredity involved the blending of traits from both parents [54]. This was incompatible with his model of evolution, which led to some disaffection with the theory by the beginning of the 20th century [55]. In contrast, the now-accepted view of heredity derives from his contemporary, Gregor Mendel (1822–1884), who detailed the persistence of characteristics from one generation to the next, now termed ‘genes’ [56]. It was not until almost the mid-20th century that Mendel’s genetics and Darwin’s evolution were merged to formulate the ‘modern synthesis’, now known as Darwinian evolution [55,57,58,59].

For us, it seems that the term ‘life’ is most appropriately used as an attribute or character of a community rather than a feature associated with any specific entity [60]. The essence of these communities is embodied in their genetic cores, an abstraction of their physical structures into a sequence of symbols which can be transmitted to the next generation in the form of nucleic acid sequences. Thus, a suitable working definition of ‘life’ seems to be ‘a self-sustaining community capable of transmitting information from parents to progeny’. ‘Transmission to progeny’ captures the propagation inherent in Darwinian evolution, and the information transmitted is the genetic core required to produce the progeny.

In essence, life is predicated on evolution and evolution requires propagation. The only class of molecules capable of self-propagation are RNAs. Proteins require RNA for propagation and DNA requires proteins for propagation, as do lipids and carbohydrates. Thus, from the advent of the first self-propagating RNA until the development of protein synthesis, life is dominated by RNA: an RNA world.

Our analysis centers on the transmission (propagation) of information from one generation to the next, parents to progeny, and thus focusses on the genetic core and its evolution, but we note that there is an essential quotient of information contained in the structure of the system. This ‘component’ is more difficult to localize than nucleic acid sequences, yet it is critical and has not been as well explored.

Life can also be viewed as the development of catalysts. Transition metals provided the first catalysts, pre-existing the biotic world. They are efficient, but have relatively poor specificity. RNA catalysts, ribozymes, are the only self-propagating catalysts, but they are relatively inefficient and have only mediocre specificity. However, ‘in the land of the blind, the one-eyed man is king’. Enzymes are the ultimate catalysts with exquisite efficiency and specificity. However, their advent required the development of protein synthesis, a complicated and intricate process. Not surprisingly, a ribozyme capable of joining amino acids into a peptide chains, peptidyl synthetase, would be of great value early in the process of evolution and has been retained as the core of the ribosome to this day.

We also note that an appropriate definition of life depends on the context. For example, from a metabolic perspective, life may be seen as a complex coordinated system of reactions, while thermodynamically life can be viewed as ‘way stations’ in the massive flow of energy as it passes from the sun or Earth to outer space [61].

Our survey begins with the transition from inanimate chemical reactions to the development of a living system, defined by the emergence of genetic information that is transmitted to progeny. We identify key innovations facilitating this development from first life to the eukaryotic cell and begin with a short review of the surprising history of the development of our understanding of heredity.

## 2. The Emergence of the Modern Synthesis

Early in the 20th century, Darwin’s idea of natural selection was melded with Mendel’s concept of the gene to produce the modern synthesis, the foundation of evolutionary theory as we know it today [62]. The search for the molecular basis of the gene began at about the same time, with proteins as the odds-on favorite. Protein’s fundamental role in living systems was well established, and with over twenty different amino acids they appeared to have the complexity a genetic core would require [59,63,64]. In 1944, Oswald Avery (1877–1955) and coworkers definitively identified DNA as the genetic material; however, this did not immediately override the prejudice in favor of proteins [65]. Then, in a short publication in 1953, James Watson (1928–) and Francis Crick (1916–2004) proposed a structure for DNA, which immediately suggested the mechanism by which nucleic acids might transmit information [66]. The suggested mechanism for propagation solidified DNA, nucleic acids, as the genetic material. Nucleic acids are unique in their ability to form long complementary duplexes, the basis for copying and propagating the genetic core. Humans have devised many ways of copying and transmitting information; however, in the natural world, nucleic acid base complementation provides the primary means of information propagation. The pairing of complementary strands allows DNA or RNA strands to produce copies ad infinitum.

Shorty after the identification of DNA as the genetic material, Crick proposed a ‘Central Dogma’ for the expression of DNA, ‘*once information has passed into protein it cannot get out again’*, which was the dominate perspective of molecular biology during the last half of the 20th century [55,67]. The first major challenge to the Central Dogma occurred when Howard Temin (1934–1994) and David Baltimore (1938–) independently observed the transfer of information from RNA to DNA via reverse transcriptase [68,69]. Initially identified in viruses, reverse transcriptase has been found in virtually all cells including eukaryotes. While, technically, this did not contradict the Central Dogma, it indicated that there was a broader range of mechanisms that can alter DNA than initially thought.

Another unexpected facet of molecular genetics is the frequent transfer of genetic information (DNA) between species: lateral gene transfer. In lateral gene transfer, DNA is passed directly from one organism to another without having to be inherited (transmitted vertically) from parents to progeny [70]. Initially observed in prokaryotes, lateral gene transfer is found among virtually all species [71,72], and can make phylogenetic analysis of lineages challenging [73]. Thus, by the end of the 20th century, there were notable exceptions to the ‘Central Dogma’, and the true nature of the evolutionary process was still being debated [74].

## 3. Venue—What Was the Nature of the Cradles of Life?

Remarkably, the venue for the origin and development of life has not been a major topic in discussions of the origin of life until recently, despite the fact that the venue is critical because it both enabled and shaped the nature of first life. Without an appropriate venue, life would not have developed, while, with a conducive venue, the origin may well have been all but inevitable.

The essential components of a conducive venue include tiny well-protected volumes within which life’s molecules could reach significant concentrations, but a place that was also permeable to the required steady external sources of energy and raw materials and permissive of the removal of waste. Traditionally, coacervates and microspheres, macromolecular droplets that form spontaneously from aqueous mixtures of organic molecules, were thought to have provided the initial containment without the need for a membrane, an idea dating back to Oparin as early as 1922 [40]. Although coacervates (protocells) remain a popular suggested venue for life’s origin, especially in the development of biology once RNA had evolved, it is difficult to imagine how sufficient fluxes of energy and material can be made available to the interior of these isolated bubbles [75,76]. If coacervates were indeed the cradles of life, they would have had to rely on diffusion to deliver the materials required to develop pre-biotic chemistry, and that would seem to preclude them from serving as effective cradles. These difficulties greatly reduce the plausibility of coacervates as a venue for the origin and development of life, in our view.

The invocation of ultraviolet light or lightning as the source of energy that drove the formations of first organic molecules, with or without a proposed role of wet/dry cycles, is also problematic [29,77]. In part, because these energy sources are destructive of large molecules and because it is hard to see how those energies could have been transferred to the interior of life’s cradles. Ultraviolet light has been championed by John Sutherland (1962–) and others [30,37], given that it can be used to energize the synthesis of a number of important precursor biochemicals including purines and pyrimidines [29,31,32,78]. However, a suitable venue with access to ultraviolet light is difficult to imagine. Similarly, high temperatures are capable of producing polypeptides, as shown by Sidney Fox (1912–1998); however, they are also highly destructive of macromolecules [79,80]. Thus, conceiving of a suitable venue with high temperatures or exposure to ultraviolet radiation or lightning which also leads to the accumulation of organic molecules in high concentrations is very challenging [29,78,79].

### 3.1. Mid-Ocean Ridge Hydrothermal Vent Systems

With the advent of the theory of plate tectonics and resulting acceptance of Alfred Wegener’s (1880–1930) theory of ‘continental drift’ [81], it became clear that the tectonic plate boundaries, especially where new sea-floor is being created, were likely to be sites for volcanic activity which could result in hydrothermal vents. After a long search, the first hydrothermal vents were discovered in the Galapagos Islands in the late 1970′s [82]. These high temperature (>300 °C) acidic vents were called ‘Black Smokers’ due to their copious black effluent [83]. The big surprise was that these Black Smokers, which existed in a virtual biological desert, were surrounded by a vibrant community of organisms including clams, fish, oysters, and weird fleshy ‘tube worms’ [83]. Unlike all other known communities, which ultimately derive their metabolic energy from photosynthesis, the energy base of the vent communities was prokaryotes which derived their energy and materials from the vent flux. To be sure, the vent animals met their requirement for molecular oxygen by the diffusion of oxygen from the surface produced by photosynthesis, but the electron donors and carbon sources came primarily from the vents.

### 3.2. Alkaline Hydrothermal Vent Systems

Soon after the discovery of hydrothermal vents, John A. Baross (1940–) and others championed the idea that these vents would be ideal sites for the origin of life [84,85]. This suggestion was, however, countered by Stanley Miller and Jeffrey Bada (1942–) who disparaged the vents as suitable venues largely because of their high temperature and acidity [86].

Based on the discovery of ‘fossil’ vents (in rocks from the Silurian of Ireland), Michael Russell (1939–) and colleagues recognized that milder vents some distance from the sites of the seafloor spreading with alkaline flux and lower temperatures would be an attractive venue for the origin of life [87], in part because the vents were riddled with tiny, approximately cell-sized microchambers [88,89]. In 2000, modern analogs of this type of vent were discovered some distance west of the Mid-Atlantic Ridge, the ‘Lost City’ hydrothermal vent system [47,89]. The system had a cooler alkaline flux, and their vent spires, largely comprised of calcium carbonate, were called ‘white smokers” (although they did not emit smoke) [48,90]. These vents were fueled by serpentinization, a geologic metamorphic process that results from the interaction between the hot rock of the upper mantle and sea water that produces heat and is an abundant source of the electron donor H_2_ [91,92,93]. Their location some distance away from the sites of active sea floor spreading results in them having much longer lifetimes than the Black Smokers, tens of thousands of years or more. The minute size of the microchambers within the vents allows reactants and products to reach very high concentrations which promotes the formation of larger molecules, particularly polypeptides several amino acids in length and polynucleotides many nucleotides in length [94,95].

In addition, the microchambers are interconnected to varying degrees. Thus, although each microchamber could only produce a small amount of product, taken together the system could produce great quantities of macromolecules. The temperature, pH, access to the vent flux, minerals found in the walls, etc., vary from one microchamber to the next, with the microchambers on the vent surface also experiencing a significant proton gradient between the sea water and the alkaline vent flux. The connection between microchambers also varies; some are virtually isolated, while others communicate freely with the mainstream of the geothermal fluids [96].

**Figure 1:**
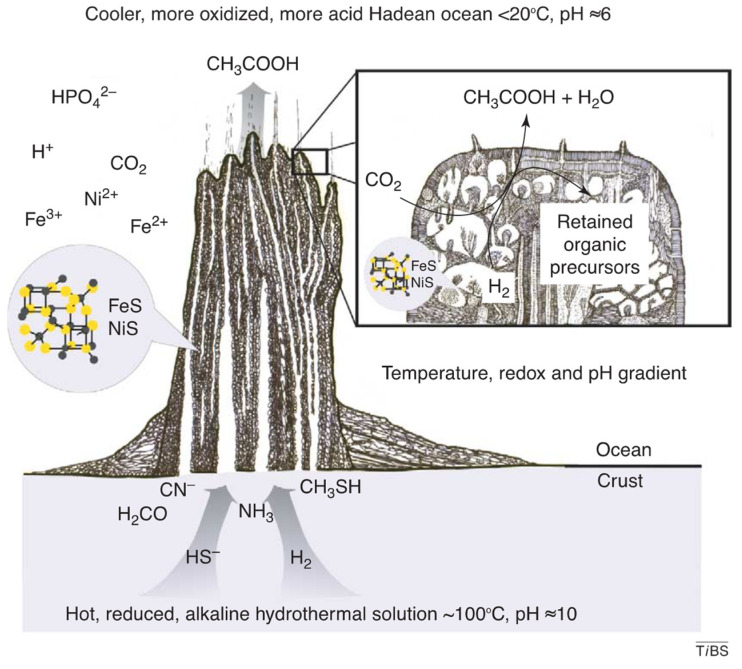
The microchamber structure and (simplified) chemistry of alkaline hydrothermal vents, reproduced from [97] with permission from the authors.

In effect, the vent microchamber system represents a vast array of simultaneously running semi-independent experiments, sharing reactants and products to varying degrees. This semi-porous system of microchambers satisfies the seemingly contradictory requirements of having small sequestered volumes, yet ready access to energy and material flows (Figure 1). The varying conditions in the various microchambers vastly expands the range of reactions that can occur and the range of products produced. In short, the microchambers of alkaline hydrothermal vent systems meet all the basic requirements for a venue for the origin and development of life [94,95]. This type of hydrothermal vent system is the most attractive venue for the origin and development of life, in our opinion, and so in the text that follows, we will simply assume that these were the cradles of life.

Note that most discussion of the alkaline hydrothermal vent hypothesis for the origin of life is framed in terms of the deep-sea vents, well below the photic zone and distant from any continental material that might have been present at the time. However, the process of serpentinization is now known in many types of environments [38]. As long as the vent flux is present, it would have mattered little where the vent system happened to be, in the abyssal depths, in the shallow marine, or even terrestrially.

## 4. Stages in the Development of Life

### 4.1. Pre-Biotic Stage

#### 4.1.1. Initial RAFs

In an alkaline hydrothermal vent system, simple organic molecules will form from the hydrogen and carbon dioxide in the vent flux, catalyzed by transition metals in the microchamber walls and the vent flux [23,97]. Serpentinization provided the electron donor (H_2_) and energy to fix CO_2_ in the early vent systems [38]. Vent reactions were promoted by inorganic catalysts, likely including greigite (Fe_3_S_4_), magnetite (Fe_3_O_4_), and awaruite (Ni_3_Fe), as well as heteroatoms incorporating Co, Mg, Al, Ca, Ti, and Zr, in the vent flux and microchamber walls [98,99,100]. Fougèrite (Green Rust) may also have played a role as a microchamber catalytic site [101]. These reactions generate the backbone of carbon and energy for metabolism [102]. The products produced are similar to the intermediates formed in the Acetyl-CoA pathway of autotrophic microbes, such as formate and acetate [9,103]. The work of William F. Martin (1957–) and coworkers suggests a vibrant pre-biotic metabolism in vent microchambers is plausible. In the absence of enzymes (or ribozymes), metal complexes such as Fe_3_S_4_, Fe_3_O_4_, and Ni_3_Fe are capable of catalyzing Acetyl-CoA type reactions [9], and the fact that today enzymes with Fe-Ni cores play a major role in the Acetyl-CoA pathway strongly supports the idea that these vents were the cradles of life (following the QWERTY principle [see Section 1.1.2 above]). Note that it has recently been proposed that the planetary impact that created the moon led to the huge pool of CO_2_ upon which first life may have depended [102].

It seems likely that the initial reactions soon built into the pathways of autocatalytic chemical reactions. These are termed reflexively autocatalytic food-generated networks (RAFs), where each reaction in the network is catalyzed by a molecule that also belongs to the network and where all network molecules are produced from a small set of external ‘food’ molecules [104]. RAFs have been characterized in the metabolism of living bacteria, presumably descendants of the initial RAFs, where metals and small molecule cofactors in combination with enzymes catalyze reactions similar to those in initial versions of the RAFs [105,106,107]. The structure of the RAFs of living organisms indicates that the first RAFs preceded the origin of RNA and proteins [106], and thus the development of a sophisticated metabolism predated heredity [27,34].

Overall, the initial production of large arrays of organic molecules, although challenging, does not seem to present an insurmountable conceptual problem [108,109,110]. We surmise that the initial reactions occurring in the vent microchamber system built into RAFs without the assistance of ribozyme or enzyme catalysts [106]. These pre-biotic RAFs were probably not as complex as modern biochemical pathways and, lacking enzyme or ribozyme catalysts, were relatively slow and inefficient. However, they would have provided a rich variety of organic molecules from which living systems could be constructed [96,97,111]. The system of developing RAFs was supported and driven by the robust source of energy and materials via the vent flux.

#### 4.1.2. Proteinoids

We presume that among the initial molecules produced in the microchambers were polypeptides of random sequences, proteinoids, given the ease with which amino acids are formed and combined [79]. Proteinoid complexes composed of various amino acids can assume a remarkable variety of configurations, and we suspect they played a wide variety of roles from functioning as (somewhat amorphous) structural elements, lining the microchamber walls (along with lipids), to facilitating the binding of smaller molecules, thus allowing them to interact with other small molecules [109]. They may also have provided hydrophobic niches with appropriate pHs where the polymerization of nucleotides and amino acids could have occurred. As with all polymers, the longer they are the more effective they are, but the spontaneous formation of polypeptides is not straightforward, so longer proteinoids were progressively rarer. Nonetheless, we surmise that even relatively short polypeptides were valuable and an integral part of the pre-biotic chemistry [111]. Once ribozymes became available, a ribozyme that could join amino acids, a peptidyl transferase, would be of great value in producing long proteinoids, even though they had random sequences. The current heart of the ribosome, peptidyl transferase, likely had a very early origin.

#### 4.1.3. Polynucleotides

**Figure 2:**
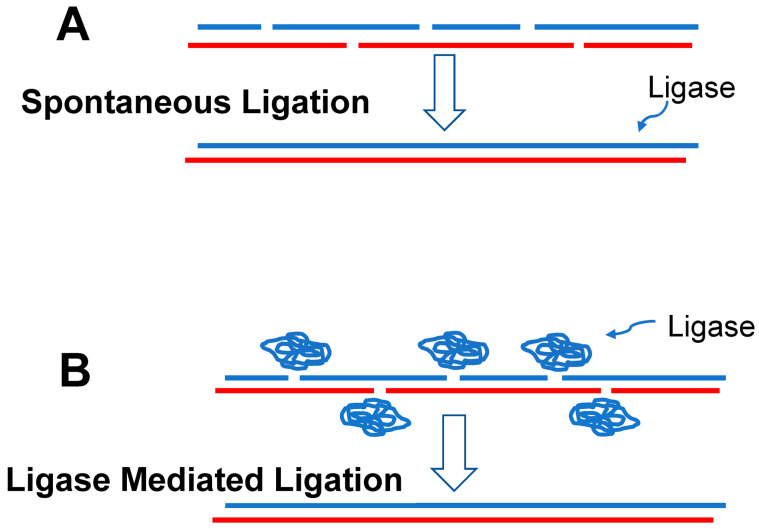
(**A**) It seems likely that the first ribozymes were generated by chance through the hybridization and spontaneous ligation of RNA oligonucleotides (blue and red strands) which produced an RNA ligase (blue strand). (**B**) With the evolution of a ribozyme capable of ligating RNA oligonucleotides (folded blue strand), production of ribozymes would have been greatly accelerated.

Polynucleotides (e.g., RNA) composed of only four different nucleotides are much less versatile than polypeptides in their three-dimensional shapes and are thus much less versatile in the functions they can assume. However, they can form duplexes with the bases in one strand pairing with complementary bases in the adjacent strand. Short duplexes could be knitted together into long strands (Figure 2), which were among the most valuable elements in the later developing RAFs due to their ability to store and transfer information [108].

#### 4.1.4. Energy Storage Molecules

In the initial RAFs the energy from one reaction could only drive another reaction in very close physical proximity. Thus, the development of intermediate energy transfer molecules was a major innovation early in the emergence of RAFs, effectively enabling action at a distance [96,111]. In modern biology, nucleotide triphosphates fulfill this role, particularly adenosine triphosphate (ATP). Acetyl phosphate (AcP) may well have been a precursor of ATP [112]. Related molecules, nicotinamide adenine dinucleotide (NAD) and flavin adenine dinucleotide (FAD), play a similar role, storing energy by adding a terminal phosphate bond. These intermediate energy storage molecules allowed energy to be created in reactions remote in time and space from the reactions where the energy would be ultimately used. This permitted the development of even more complex reaction networks in which the energy could be shuttled among reactions [110].

With time, we surmise that RAFs increased in size and complexity, but they could not reproduce [113] as they had no genetic core. Thus, the particular properties of any given RAF are the product of their generating environment and simply reflect its parameters and conditions—although the pre-biotic RAFs likely became very complex and sophisticated; lacking a genetic core, they remained solely the product of their environment, unable to evolve.

### 4.2. RNA and First Life

Historically, with DNA accepted as the (only) genetic material, ‘the mother of all’ conundrums arose relative to the origin of life; DNA is necessary to make proteins, but proteins are required for the copying of DNA. So no-one could see how life could have started. In 1982, this conflict was resolved by the discovery of ribozymes, catalytic RNA molecules [114,115]. RNA is capable of both transmitting genetic information and catalyzing reactions; the existence of an RNA capable of catalyzing its own replication solved the conundrum. This positioned RNA as the transition molecule bridging the non-genetic pre-biotic RAFs and the genetic biotic world. If the transition from non-life to life could be pinpointed to a single step, it would be the origin of an RNA ligase, the advent of heredity. These RNA ligases were able to replicate themselves as well as all other RNAs. It has also been proposed that heredity developed through interactions between RNA and short polypeptides [25,26,27].

David Bartel (1961–), Jack William Szostak (1952–), and others have made substantial progress in experimentally generating ribozymes [116,117]. Gerald Joyce (1956–) and coworkers have been able to produce an RNA ligase capable of self-replication in vitro [118]. Marshall and Brunk call this phase in the development of life the ‘plus RNA World’ to highlight the fact that ribozymes probably evolved in an already sophisticated prebiotic system [34], including a wide range of molecules large and small as well as surface catalysts. Given the prior existence of these catalysts, the fundamental innovation represented by RNA is the advent of heredity.

In modern biology, RNA is synthesized by an RNA polymerase that adds a single nucleotide at a time to a growing RNA chain hybridized to a DNA template. However, we suspect that the first replicating RNAs most likely formed via the hybridization of oligonucleotides, short strings of single-stranded RNA (Figure 2), facilitated by the molecular complexity within the microchambers [22].

While the RAFs were solely a function of their environments, with the advent of RNA replication, information could be transmitted independent of the environment. The variation in the information transmitted is the basis for selection and the system would thus evolve. The advent of evolving ribozymes rapidly transformed the initial RAFs into sets of ribozyme-catalyzed reactions which enhanced their speed and permitted their specificity to be refined and expanded. At this stage, a fully functional biological system came into existence.

While RNAs were the molecular basis of both catalysis and replication, driving the evolution of all types of RNAs, other macromolecules likely played critical roles, particularly proteinoids. A ribozyme capable of forming proteinoids, that is, forming peptide bonds (a primitive peptidyl transferase), would have been invaluable at this stage of life’s evolution.

The fact that the peptidyl transferase at the center of modern ribosomes is still a ribozyme provides support for an early role of RNA catalysis [119,120,121]. Given that protein catalysts (enzymes) are so much more effective than RNA catalysts (ribozymes), it is perhaps surprising that the ribozyme peptidyl transferase activity was not replaced by an enzyme after the development of protein synthesis. The probable explanation for the persistence of a ribozyme in this role is that peptidyl transferase activity was so crucial that it was not possible to swap the ribozyme out for an enzyme, in the same way that swapping out helicopter blades during flight is hard to envision—the relatively slow-acting peptidyl transferase is ‘baked in anachronism’, reflecting a key early step in life’s evolution following the QWERTY principle.

With evolving ribozymes, it is likely that a system of biochemical pathways evolved. In addition, many ionic molecules were already present in the vent flux and lined the microchamber walls, including green rust (fougérite), enhancing the biochemical pathways [101,122]. It seems likely that proteinoids, polysaccharides, lipids, and other macromolecules combine with the replication of RNAs to produce quasi-cells, vent creatures, within the microchambers. The rocky walls of the microchambers were most likely lined with proteinoids and other macromolecules that regulated molecular entrance and egress. The concentration of organic molecules in the microchambers could rise to exceptional levels, and a wide variety of molecules were probably shared among the microchambers.

### 4.3. Protein Synthesis

The next and dominant event in the development of life was the advent of protein synthesis. In the modern cell, the immense variety of proteins, not only enzymes but also critical structural and regulatory elements, largely define biology; proteins are the quintessential biological molecule. In the modern ribosome, although the peptidyl transferase is still a ribozyme, there are some 50 ribosomal proteins in addition to a smaller set of mostly much larger RNAs. The ribosomal proteins are not catalytic, but provide structural stability to the massive rRNAs, not unlike knitting needles in a ball of yarn. Reuveni et al. (2017) provide an analysis of the selective forces responsible for the protein and RNA composition of the ribosome [123]

Protein synthesis is a remarkable and complex process that translates with high fidelity the nucleotide sequences carried in the DNA into proteins’ sequences of amino acids [124]. In outline: DNA sequences are transcribed into messenger RNA (mRNA) sequences; the mRNA is then translated into a protein sequence using transfer RNAs (tRNAs), where one end of the tRNA corresponds to the one of the codons of the genetic code, while the other end binds the corresponding amino acid [125]. The tRNAs, charged with appropriate amino acids, hybridize with the mRNA while the peptidyl transferase ribozyme adds the attached amino acid to the growing protein by forming a peptide bond. Each specific tRNA is charged, bonded to the appropriate amino acid, by a specific amino acyl-tRNA synthetase enzyme.

The translation of the mRNA sequence of nucleotides into a sequence of amino acids requires a ‘genetic code’ relating to a specific codon, a series of three nucleotides, to a specific amino acid. There are 64 codons in the genetic code, and each one specifies one of the twenty amino acids (or serves as start or stop codons) [126].

The steps by which the complex process of protein synthesis developed are yet to be understood [126], but see also [25,26,27]. The development of a complex process such as protein synthesis is greatly aided by a system with a myriad of simultaneous relatively independent experiments all contributing to the final process. The vent microchambers provide such a system.

The traditional view, stemming from the co-dependence of DNA and proteins in modern cells, is that the biology (e.g., catalysis) of first life was dominated by RNAs (ribozymes in the absence of protein and DNA), the so-called RNA world; see [115,127,128] for a review. With the development of protein synthesis, specific proteins could be generated with ease. The replacement of ribozymes by the much more specific and efficient enzymes probably ensued rapidly, leading to networks of biochemical reactions very similar to those found in modern organisms. The RNA-world hypothesis suggests that modern biological function emerged via the piecemeal replacement of ribozymes by enzymes. However, while we would not be surprised if many ribozymes were replaced with a functionally equivalent enzyme, it is also possible that entire ribozyme-catalyzed pathways were replaced with entirely new enzyme-catalyzed pathways. That is, the biology of ribozyme-catalyzed proto-life may have been quite different from the biology of enzyme-catalyzed life, in the same way that a vehicle driven by an internal combustion engine is very different to a vehicle driven by muscle, or by jet or rocket engines.

### 4.4. Exploiting the Natural Proton Gradient to Augment ATP Production

Up to this point, the energy driving the entire system was provided by the vent flux [111]. However, the natural proton gradient developed between the (at the time) slightly acidic ocean and the alkaline vent flux represented a significant potential energy source, particularly for the outer microchambers of the vent system which bordered on the ocean [97]. In the 1960′s, Peter Mitchell (1920–1992) proposed the chemiosmotic hypothesis, which posited that the movement of protons across a membrane could generate ATP [129]. At the time, his hypothesis was viewed as somewhat bizarre, but was confirmed by the detailed analysis of ATP synthase by Paul Boyer (1918–2018). ATP synthase is a remarkable molecular machine involving a membrane-bound stator surrounding a rotor which turns as protons cross the membrane and pass through the machine [130]. This elaborate and complex mechanism is apparently the preferred means of generating ATP as it is employed by virtually all living systems. The link between the natural proton gradient of the vent microchambers and how ATP is generated today via a (now internalized) proton gradient provides strong support for the role of hydrothermal vents in the origin of life [111,131].

However, interior microchambers would not have had access to the natural proton gradient. Thus, after the evolution of ATP synthase, there would have been a selective advantage for interior vent creatures to generate a proton gradient internally. This is what the electron transport chain does, passing protons across membranes as electrons are passed from one complex of the transport chain to the next [131,132]. The advent of the electron transport chain allowed all the microchambers to use ATP synthase to produce ATP.

### 4.5. DNA

The differences between DNA and RNA make DNA significantly more chemically stable than RNA, but also less flexible, preventing it from forming catalytic conformations. This suggests that DNA as an information storage molecule may have been a later innovation, eventually replacing RNA as the primary genetic core material, even though DNA may have arisen at approximately the same time as RNA [22]. The fact that the mechanisms of DNA replication in bacteria and archaea are different [133] suggests the possibility that DNA, as the primary information storage molecule, evolved independently in each of these lineages [133,134,135].

## 5. Cellularization and Life beyond the Vent

We think all the developments up to this point took place in a vent microchamber complex [95]. The microchamber rock walls provided stability and shelter, but they were most likely lined with elaborate membrane-like structures that evolved to modulate the entrance and egress of molecules [89]. Periodically, the walls of microchambers on the vent outer surface would have broken down, releasing their contents. Early on, these contents were simply lost to the surrounding ocean, but as the membrane linings of the microchamber walls matured, the released outermost vent creatures would have been able to persist. At some point, they were probably able to exist independent of their microchamber cradles, and stable cells emerged from the vent chimney surfaces.

However, newly released cells would have been essentially cut off from the energy and material flows provided by vent flux—the region surrounding the vent was a virtual desert [82]. Thus, newly released cells would likely only have found a sustaining source of energy and materials if they remained within the vent outflux stream. Hence, they initially probably remained close to the microchambers, or even attached to them. This supposition gains support from the observation that the independently evolved locomotory organs of the bacteria (the flagellum) and archaea (archaella) both have at their core adhesins, molecules that enable the attachment of the cell to external surfaces [136]. Locomotion evolved later, built upon these adhesin cores.

The new free-living cells traded the security of the microchambers with their sustaining vent flux for mobility and the potential to explore the whole Earth. The plausibility that free-living cells could have arisen in this manner seems reasonable, but understanding how they moved beyond the exit stream of the vent flux is more problematic. However, as the molten mantle solidified within the early Earth, it is quite possible that hydrothermal activity driven by serpentinization may have covered much of the sea floor by the middle of the Hadean [137]; the oceanic seafloor, when life originated, may had been replete with useable redox gradients as resources for the first cells.

### 5.1. At Least Two Cellularization Events

Initially, prokaryotes were lumped into a single category called bacteria, even though they had a variety of shapes, metabolisms, and lifestyles. In 1977, using partial rRNA sequences, Carl Woese (1928–2012) determined that there were two distinct lineages of prokaryotes, bacteria and archaea, which are different to each other, as each is to eukaryotes [138]. Essentially, all cells share the same detailed genetic code and the same exact transcription and translation machinery that converts DNA information into proteins. These systems consist of dozens of similar proteins and RNAs, indicating they had a common ancestry through the development of protein synthesis. On the other hand, they differ significantly in their metabolic biochemical pathways. Further, bacteria have ester-linked membrane lipids while archaea membrane lipids are ether-linked. Archaea also have pseudopeptidoglycan and glycoprotein in their cell walls, while bacteria typically use peptidoglycans. These differences between bacteria and archaea most probably arose before these linages embarked on a free-living existence outside the vent system of their origin, as cell walls and membranes are essential for free-living cells. Similarly, the highly complex locomotory nanomachines of bacteria and archaea are made from completely different sets of molecules, indicating that they evolved independently [136]. The evidence thus points strongly to these differing lineages emerging independently from the same microchamber complex.

The existence of two progenitor prokaryotic linages is unexpected. A priori, the emergence of just one lineage from a single progenitor vent, or multiple linages from different vents, would seem more likely, rather than two differentiated lineages emerging from a single ‘origin vent’. A potential explanation for the dual origins of autonomous cells from a single ancestral vent is the fact that the vent complexes were probably vast. For example, the largest vent chimney at the Lost City site (Poseidon) is 60 m tall and some 100 m across. With ~10^11^ microchambers/m^3^ (assuming each is ~100 microns across), that comes to some 10^17^ microchambers in that one chimney, an astronomical number of microchambers.

### 5.2. The Diversification of Electron Donors and the Origin of Photosynthesis

Upon their emergence, the earliest prokaryotic lineages likely formed communities in the immediate vicinity of the vent fluxes, analogous to the community of organisms found surrounding modern vents. Once free of the vent microchambers, different lineages were able to adapt to the broad range of electron donors likely available [139] and beyond the hydrogen they relied on within the vents, expanding the range of environments that they were able to live in.

We do not know the water depth of the ‘origin vent’, but presumably, statistically, it was likely below the photic zone. It is even possible that the planet’s crust in the Hadean was completely submerged by an ocean to perhaps a depth of 5 km [36,140]. Alternatively, it is possible that there was extensive surface crust even this early in the Earth’s history [141]. Regardless, at some point, life reached the photic zone, and may even have evolved within the photic zone.

Once in the photic zone, an immense energy source in the form of solar energy became available. The development of photosynthesis (photosystem I) ensued with solar photons exciting electrons from reaction centers in molecules like bacteriochlorophyll to split hydrogen sulfide, producing sulfur as a byproduct [24]. The excited electrons tumbled down a modified version of the electron transport chain, producing ATP and nicotinamide adenine dinucleotide plus hydrogen (NADH). These photosynthetic bacteria were autotrophs, capable of producing complex organic compounds from carbon dioxide and hydrogen powered by solar energy. An additional set of reactions termed the Calvin cycle utilized the ATP and NADH to convert carbon dioxide and water into organic compounds including glucose [97,107].

Following the original development of photosynthesis, two versions of the original photosystem, called photosystem I and photosystem II, were coupled, enabling solar photons to split water (instead of hydrogen sulfide), producing molecular oxygen and hydrogen [142]. The excited electrons from photosystem II are fed into photosystem I to produce more ATP and NADH. This two-phase photosynthesis has the distinct advantage of being able to use water as the electron donor, which is vastly more available than hydrogen sulfide; thus, bacteria could become global in their distribution.

However, the byproduct of splitting water is molecular oxygen, which is highly reactive, readily oxidizing most compounds, and thus presented a major hazard. The toxicity of molecular oxygen was ameliorated by ‘burning the trash’, oxidizing the waste products of metabolism such as lactate and ethanol. The oxidation of these wastes protected other compounds while generating a great deal of energy. A process called oxidative phosphorylation (or electron transport-linked phosphorylation) evolved to utilize this energy, producing ATP, NADH, and flavin adenine dinucleotide (FAD). Thus, in the process of ameliorating the deleterious effects of molecular oxygen, a significant additional source of energy was created.

At first, the molecular oxygen generated was localized to the photosynthetic bacterial communities, but eventually it spread to the Earth’s oceans and atmosphere, oxidizing these environments in the process. This was the most profound change on Earth ever produced by living systems. The conversion of the Earth’s atmosphere to an oxidizing state took a long time, as there was an enormous amount of material to be oxidized before oxygen levels could build up. These oxygen sinks include copious amount of Fe^2+^ in the ocean, which upon oxidation led to the vast iron oxide deposits found in rocks of about 2.5 billion years old. An oxygenic atmosphere permitted the development of aerobic organisms, including the vast majority of eukaryotes and most modern prokaryotes as well.

**Figure 3:**
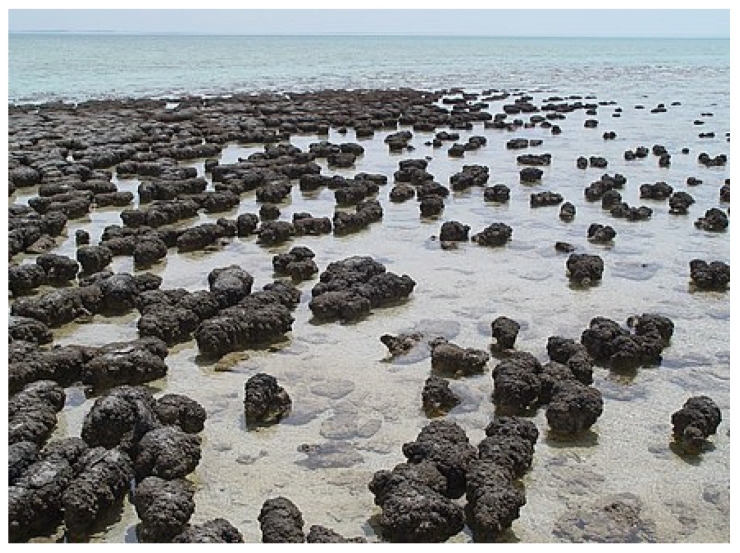
Modern stromatolites growing in Shark Bay, Western Australia. These stromatolites are roughly an equal mixture of minerals and cells, including both bacteria and archaea. The outer surface of stromatolites became a haven for oxygenic photosynthetic bacteria; thus molecular oxygen was available here long before it was prevalent in the atmosphere. Image courtesy of Paul Harrison licensed under GFDL.

Within the photic zone, it is likely that complex communities of photosynthetic bacteria and archaea (which are not photosynthetic) formed stromatolites like those shown in Figure 3.

Fossil stromatolites provide our earliest evidence of cellular life, [143]. These stromatolites are roughly an equal mixture of sand (rock) and cells, including both bacteria and archaea with interiors that tend to be anaerobic, favoring archaea, although bacteria generally dominate throughout. Stromatolites are usually found in shallow water, well within the photic zone, where solar energy is abundant. The outer surface of stromatolites became a haven for oxygenic photosynthetic bacteria; thus, molecular oxygen was present here long before it was prevalent in the atmosphere. From their aerobic surface to their anaerobic interiors, stromatolites provide an ideal venue for the development of both aerobic bacteria and anaerobic archaea, as well as, ultimately, eukaryotes.

## 6. Prokaryotic Cells versus Eukaryotic Cells

From the earliest microscopic examination of cells, the similarity of mitochondria and chloroplasts to bacteria was noted. Lynn Margulis (1938–2011) became a champion of the symbiotic formation of eukaryotes from the time she was a junior faculty member [144]. She avidly promoted the idea that the mitochondria found in eukaryotic cells were of aerobic bacteria origin that had been ingested by a large anaerobic cell: symbiogenesis [145,146]. The chloroplasts found in plants were similarly ingested photosynthetic bacteria as well [145]. Margulis did not limit her proposal of symbiosis to mitochondria and chloroplasts; she also claimed that virtually all eukaryotic cells’ organelles were the product of symbiosis, including the nucleus, cilia, flagella, and others [146]. DNA sequencing confirmed the origin of mitochondria and chloroplast to be bacterial; all the other organelles apparently arose from within the eukaryotes.

One of the major early hypotheses for the origin of eukaryotes proposed that a large precursor to the eukaryotic cell lacking mitochondria, but capable of phagocytosis, engulfed the bacteria, which were to become the mitochondria [147]. However, this idea has been largely abandoned, as there is no evidence or plausibility for a large anaerobic phagocytic prokaryote.

Eukaryotes are generally much larger and more complicated than prokaryotes. A typical prokaryote is 1–2 µm in diameter, while eukaryotes are usually 20–50 µm, thus the volume of a eukaryotes is 1000–125,000 times the volume of a prokaryote. In addition, eukaryotes have elaborate internal structures, including extensive membranes and organelles (mitochondria, chloroplasts, nuclei, endoplasmic reticulum, Golgi apparatus, etc.). Although prokaryotes are fascinating in their own right, the complexity of eukaryotes clearly involves mechanisms and pathways far beyond those found in prokaryotes; perhaps unsurprisingly, only eukaryotes gave rise to sophisticated multicellular organisms.

At a genetic level, bacteria and archaea are also vastly different to eukaryotes. In bacteria and archaea, genomes vary but are modest in size. A typical bacterium has about 3 to 5 Mb (million base pairs) and encodes around 5000 different proteins. Eukaryotic genomes also vary, but are generally much larger, typically of about 3000–5000 Mb, encoding up to 100,000 different proteins.

The organization of the prokaryotic genome is simply a single circular DNA molecule, while the eukaryotic genome is very different. Eukaryotic DNA is organized into linear chromosomes composed of chromatin facilitated by histones, DNA binding proteins that form regular compact structures [19]. To be sure, proteins are affiliated with prokaryotic DNA, but there is no regular structure.

Prokaryotic DNA is usually attached to the cell wall and is parceled out to the daughter cells when the cell divides. In eukaryotes, the chromosomes are directed to the daughter cells during cell division by a complex mechanism, mitosis, involving specialized contractile proteins.

Within the prokaryotic genome, upwards of 80% of the DNA codes for proteins, with much of the remaining genome involved in gene regulation. In contrast, only a small fraction of the eukaryotic genome codes for proteins, less than 3% in that of humans. The remainder, and the vast majority, of the eukaryotic genome’s DNA has been characterized as ‘junk DNA’ [148]. However, in 2003, the Encyclopedia of DNA Elements project (ENCODE) was launched to build a comprehensive list of functional elements in the human genome [149]. ENCODE found that about 80% of the human genome is transcribed, which suggests that it is functional. There was significant push back, noting that transcription does not automatically assure function [148,149,150,151], but transcribing that much DNA strongly suggests some function. We now have a much more sophisticated understanding of the remarkable biology of RNAs [152,153].

**Figure 4:**
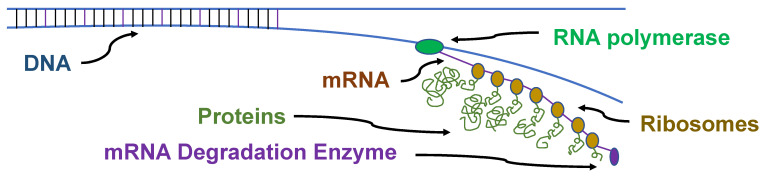
Prokaryotic coupled transcription and translation. The DNA is transcribed into an mRNA which is immediately translated into proteins by multiple ribosomes. Frequently, a polycistronic mRNA begins to be degraded before the mRNA is completely transcribed.

Gene expression in prokaryotes is also very different than in eukaryotes. Prokaryotic genomes have many polycistrons, genes for a set of related proteins that are directly adjacent in the genome and transcribed as a single mRNA (Figure 4). Transcription and translation in prokaryotes is frequently coupled, where the translation of an mRNA begins before transcription is complete, especially for the polycistronic mRNAs. The degradation of the mRNA can begin before the transcription of the genes is complete.

**Figure 5:**
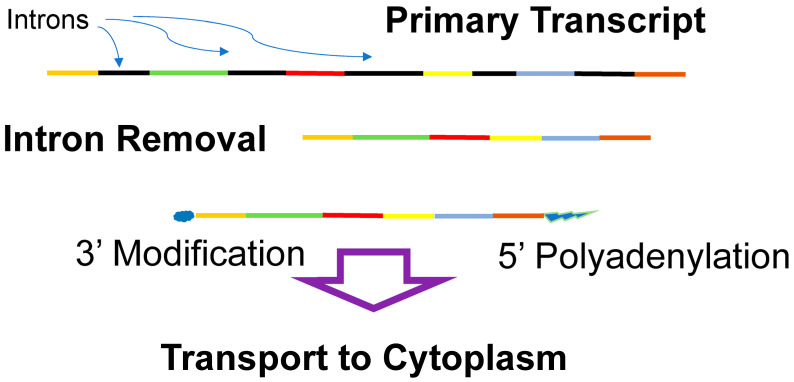
Eukaryotic mRNA processing. The primary transcript of eukaryotic genes contains exons (where the identity of the exons is indicated by the different colors) and introns (black), which are removed. The final mRNA has its 3′ end modified (methylation), and its 5′ end is polyadenylated. The processed mRNA is then transported to the cytoplasm for translation.

In eukaryotes, transcription occurs in the nucleus and translation takes place in the cytoplasm; thus, coupled transcription and translation is not possible. Instead, the eukaryotic mRNA is extensively processed after transcription, prior to transport to the cytoplasm (Figure 5). Eukaryotic genes frequently have introns (non-coding regions) interrupting the coding regions (the exons), which must be removed prior to translation. After transcription, the introns (non-coding regions between the exons) are spliced out (excised). The function of introns is unclear; however, they may play a role in gene regulation and almost certainly accelerate the generation of novel proteins as exons can be joined in alternative arrangements (alternative splicing). In addition to the removal of introns, the processing of the eukaryotic mRNA includes the methylation of the 3′ end (capping) and the addition of a polyadenosine chain to the 5′ end. Segments of a eukaryotic gene may also be rearranged during mRNA processing, alternative splicing, which allows approximately 20,000 coding regions (genes) in the human genome to give rise to well over 100,000 different proteins [154]. The modified eukaryotic mRNA is then transported to the cytoplasm, where it is translated. This elaborate manipulation of mRNA affords numerous opportunities for gene regulation both in the nucleus and in the cytoplasm.

## 7. Moving beyond a Gene-Centric View

During the first decades of the 21st century, a vast amount of data on gene expression, particularly in eukaryotes, has accrued. It is clear that there is a great deal of regulation of gene expression superseding the simple ideas of the ‘central dogma’ and the ‘modern synthesis’ [155]. The gene-centric perspective of the 20th century appears to be inadequate in view of this additional information [156,157]. An expanded perspective is frequently referred to as the ‘extended evolutionary synthesis’, representing a shift from a ‘bottom up’ gene-centric perspective to including a ‘top down’ physiological function-oriented perspective [34,74,158].

Genes apparently work through the intricate and complex interactions of interconnected pathways. When an enzyme in a major pathway is disabled, ancillary pathways become more fully engaged, so that the function of the effected pathway is only slightly curtailed, if at all. The concept of ‘one gene, one function’ is simply untenable for eukaryotes. Additional data also indicates that the environment plays a significant role in gene expression. The feedback of information from environmental conditions transmitted to the inherited genetic core (the DNA) is a direct challenge to the Central Dogma. However, increasingly credible evidence suggests something like this may occur [109,157,159,160]. Environmental conditions provide feedback that alters gene expression in such ways that a unidirectional flow of information from the genome to the environment is not tenable [158]. The ‘inheritance of acquired characteristics’ has been suggested, although few mechanisms have been proposed, and there is no evidence for the modification of the genetic core (DNA sequence) based on acquired characteristics [157,159].

## 8. The Origin of Eukaryotic Cells

**Figure 6:**
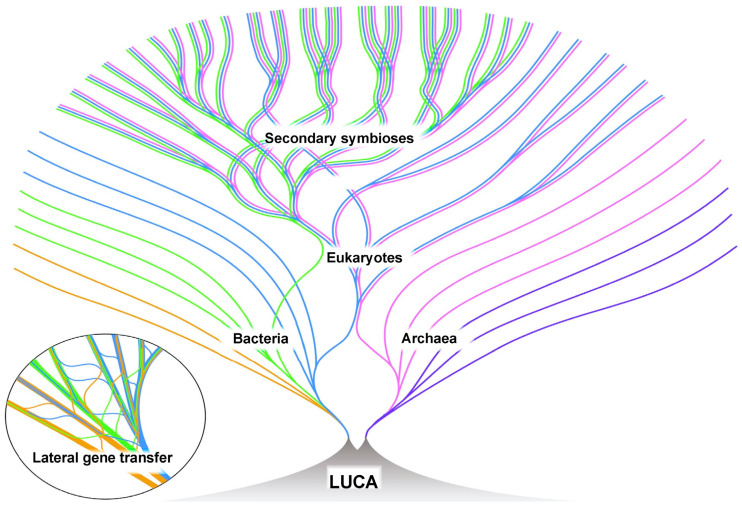
Living linages of bacteria, archaea, and eukaryotes are depicted over time (toward the outer portion of the diagram). LUCA, the Last Universal Common Ancestor of these living species, is depicted as existing in a vent system prior to the emergence of bacteria and archaea. There was an extensive lateral gene transfer between lineages, particularly those bacterial and archaeal (insert). Eukaryotes resulted from the merger of a bacterium and an archaeon [161]. After the development of eukaryotes, secondary symbioses led to photosynthetic eukaryotes. Many lineages likely became extinct over time, but are not depicted. Figure reproduced from [21] with permission from the author.

In our opinion, the rise of eukaryotes is best explained by ‘The Hydrogen Hypothesis for the First Eukaryote’ published in 1998 by William Martin and Miklós Müller (1930–) [161]. They suggest that in an anaerobic environment, an archaeon was attracted to a bacterium by the hydrogen waste produced by the bacterium, possibly in a locale such as the interior of a stromatolite. Over time, this affiliation became more pervasive, with the archaeon ultimately surrounding the bacterium. The bacterium was capable of aerobic respiration, and ultimately became the mitochondria of the developing eukaryotic cell [21]. The genetic core in the emerging nucleus became organized into chromosomes built around histone proteins provided by archaeon [19] and contains a mixture of genes from the archaeal partner and genes transferred from the developing mitochondria (bacterial partner) [21,161,162]. The mitochondria continue to replicate within the cells as semi-autonomous organelles. The bacterial partner provided the energy source (oxidative phosphorylation) and contributed membranes for the development of the extensive internal structures characteristic of eukaryotes. Through the reorganization of the biochemistry from both the bacterial and archaeal partners, the chimera began to function as a single unit. Later, the symbiotic incorporation of photosynthetic bacteria by some eukaryotic cells produced chloroplasts. Like the mitochondria, chloroplasts also exist as semi-autonomous organelles. The overall development of eukaryotes is depicted in Figure 6.

## 9. The Deep Evolutionary Heritage of the Eukaryotic Cell

Finally, we wish to emphasize the key evolutionary events in the origin of the eukaryotic cell that predate its origin. The power of eukaryotes comes from oxidative phosphorylation, whose origin was a response to the production of molecular oxygen enabled by the coupling of photosystem I and II in a different bacterial lineage’s response to living in the photic zone. Photosynthesis itself relies on the electron transport chain, which in turn evolved in response to the opportunities offered by internalizing the external proton gradient, which played a role in the development of early life inside the hydrothermal vent microchambers. Thus, while the first eukaryotic cell was not photosynthetic, eukaryotes owe their origin to the evolution of photosynthesis. Similarly, the large and sophisticated genome of the eukaryote owes its origin to the development of histone-like proteins in the archaeal partner, which were a response to the selective pressure to stabilize its genome at the high temperatures it lived in. We are all products of a massively anastomosing history of seemingly unrelated innovations.

## Data Availability

Not applicable.
